# Using an automated tail movement sensor device to predict calving time in dairy cows

**DOI:** 10.3168/jdsc.2023-0445

**Published:** 2024-01-15

**Authors:** S.G. Umaña Sedó, D.L. Renaud, J. Morrison, D.L. Pearl, J.F. Mee, C.B. Winder

**Affiliations:** 1 Department of Population Medicine, University of Guelph, Ontario, N1G2W1, Canada; 2 Teagasc, Research Centre, Fermoy Co. Cork, P61 C997, Ireland

## Abstract

This study aimed to evaluate the effectiveness of an automated tail movement sensor device (Moocall; Bluebell, Dublin, Ireland) to predict time of calving in dairy cows. At a commercial dairy farm in southern Ontario, Moocall (MC) devices were attached with the device's strap, and an additional elastic wrap, to the tail of cows approximately 3 d before their expected calving date. The MC has 2 types of alarm, a high activity alarm in the previous hour (1HA), and a high activity alarm in the previous 2 h (2HA); these alarms were sent and registered to the MC software. The calving and close-up pens were video monitored to determine the exact time of the onset of stage II of calving (amniotic sac visible at the vulva) and the end of stage II of calving (total expulsion of the calf). A total of 49 cows were enrolled, but we excluded 13 animals from the analysis as they had 3 or more MC drops from the tail (n = 6), a swollen tail (n = 3), or the MC device was lost (n = 4); this left 36 cows. In total, the device dropped off 21 (42%) cows. The average number of alarms (1HA and 2HA) per cow before stage II of calving was 2.7 ± 2.3 (± standard error). The first alarm after fitting the device on the tail was used to determine the device's sensitivity and specificity. Depending on the interval before the onset of parturition (i.e., 2, 4, 8, 12 h) in which the alarm was triggered, sensitivity varied from 5% to 72% and specificity from 50% to 93%. The false positive rate varied between 6% and 50% depending on the interval from the alarm to the onset of parturition. The high false positive and device drop rates (despite the addition of the elastic wrap) may compromise the applicability of this sensor device in a commercial setting.

## Highlights

• Time of calving was best predicted from the first alarm at 12 hours before calving compared with alarms closer to calving.
• The analysis of both alarms combined was the most accurate to identify a calving event.
• A substantial number of device losses and tail injuries may compromise the utility of this device in a commercial setting.

## Graphical Abstract

**Figure:**
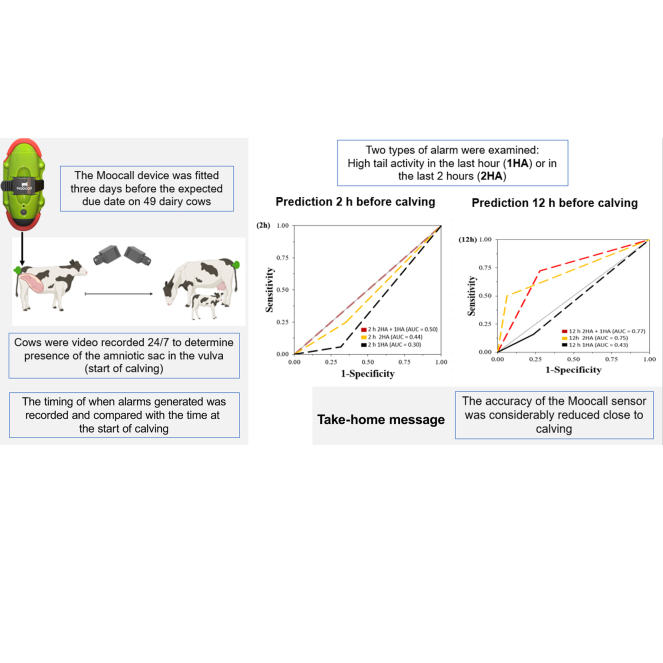
**Summary:** This study aimed to evaluate the diagnostic accuracy of an automated tail movement sensor device (Moocall; Bluebell, Dublin, Ireland) to predict calving time in dairy cows. On a commercial dairy farm in southern Ontario, 49 Holstein-Friesian cows were enrolled and had a Moocall device attached with an elastic wrap to their tail approximately 3 days before their expected calving date. High tail activity triggered 2 alarms, namely, high activity in the last hour or in the last 2 hours. Prediction of calving time within 12 hours from both alarms combined had a sensitivity of 72% and specificity of 72%. However, the high number of false positive alarms and multiple device losses may compromise the utility of this device.

Accurate prediction of calving time could facilitate timely movement of cows to the maternity pen, identification of dystocia, prevention of stillbirth and postparturient disorders, and provision of perinatal care. Historically, farmers have managed their calving surveillance around the expected calving date (**ECD**). However, genetic and environmental factors can influence the ECD by as much as ±7 d (Norman et al., 2009). Thus, ECD cannot predict the calving time and only approximately the calving day.

The assessment of timing of parturition has traditionally been accomplished by recognizing behavioral and physiological changes before calving. The first stage of calving (i.e., phase of cervical dilation) is characterized by behavioral signs such as elevation of the tail and increased locomotion and lying bouts (Barraclough et al., 2020; Giaretta et al., 2021). These have been reported to increase in frequency in the 24 h before calving and reach a peak 4 to 6 h before calving (Barraclough et al., 2020; Giaretta et al., 2021). In addition, enlargement of the udder, vaginal mucous discharge, and relaxation of the pelvic ligaments occur during the stage I of calving (Schuenemann et al., 2011). However, these physiological and behavioral changes can vary substantially from cow to cow (Berglund et al., 1987). Additionally, monitoring cows every 3 to 5 h day and at night can be challenging in herds with limited labor, especially in seasonal calving systems.

There has been a rapid growth in recent years in precision livestock technologies (**PLT**), including calving prediction devices, to compensate for a lack of availability of skilled farm labor (Crociati et al., 2022). One PLT used to detect when a cow is due to calve is a tail movement monitoring device. Tail activity (e.g., tail elevation, movements, duration) increases during the last 12 h before calving and it is considered one of the most important behaviors for predicting calving (Miedema et al., 2011; Giaretta et al., 2021). A tail movement sensor device (Moocall; Bluebell, Dublin, Ireland) was developed to measure specific tail movements that alert the producer that calving is approaching. Limited research has been done to validate the Moocall (MC); however, results have shown a low accuracy in predicting a calving event (Crociati et al., 2022). This may be a result of limited sample size (Horváth et al., 2021), a lack of definition of when calving starts and ends (Giaretta et al., 2021), or multiple (2 or more) reattachments of the MC sensor per animal, which could have altered the MC accuracy (Voß et al., 2021).

Given these limitations in existing MC research, we conducted a validation study to evaluate the diagnostic accuracy of a MC-automated tail movement sensor device for predicting the beginning of stage II of calving in dairy cows. We hypothesized that the MC's predictive ability would be constant or improve closer to the onset of stage II of calving.

This study was carried out from June 2021 to October 2021 at a commercial dairy farm, selected due to its proximity to the University of Guelph, in southern Ontario, Canada. Animal use approval (AUP #3851) was received from University of Guelph Animal Care Committee. All dry Holstein-Friesian cows were housed in freestalls that were sand-bedded. Multiparous cows (n = 49) were moved to a single freestall close-up group pen with an adjacent scrape alley approximately 21 d before their due date. Animals had water access ad libitum, and each cow was fed a TMR diet. The farm used just-in-time calving management where the farmers would move animals showing signs of stage II of parturition (amniotic sac or fetal extremities visible at the vulva) to 1 of 2 individual calving pens, each of which had a straw pack area (20.0 × 18.8 ft; 6.0 × 5.4 m) and an adjacent scrape alley (13.3 × 18.8 ft; 3.9 × 5.4 m).

The MC device consists of an encased inclinometer (16 × 8 × 10 cm, 320 g) with a strap for tail mounting and a red rubber pad to prevent slipping. The MC collects data on specific tail movements (i.e., tail raising, tail twitching) every second after activation and uses an algorithm to trigger alarms, which are then sent by SMS to a MC cellphone application (Moocall app, Moocall, Bluebell, Dublin, Ireland). Tail movements unrelated to calving (e.g., chasing off flies or raising the tail for urination and defecation) are filtered out by the MC algorithm. Five types of alarms provide information about relevant events and sensor status. The first 2 types of alarms are generated by increased frequency of tail movements for 1 h (i.e., high tail activity in the previous hour; **1HA**) and for another hour after that if tail movements continue (i.e., high tail activity in the previous 2 h; **2HA**; Moocall Calving Sensor Ireland, 2022). Other warnings from the MC device include lack of tail movements (indicating that the sensor may have fallen off), poor internet connection status, and continuous attachment for more than 4 d.

In this study, 3 d before the predicted calving date (d 275), based on a 278-d gestation length (Johanson and Berger, 2003), an MC device was attached to each cow's tail following the manufacturer's guidelines (Moocall Calving Sensor Ireland, 2022) by 2 trained graduate students; in total 9 MC devices were used. Based on the multiple drops of the MC device reported previously (Voß et al., 2021), an elastic wrap (3MVetrap, St. Paul, MN) was placed around the MC device to further secure it to the tail. If the MC software detected an MC device to be off the tail, it was generally refitted within 3 h of the dropping event by the trained graduate students. Data from the MC cellphone software were only received and stored by a trained graduate student; farm personnel did not have access to the MC software or the MC devices. Cows were video recorded in both the close-up pen and in the calving pen (2.8 mm Outdoor Analog Dome Camera, Hikvision, Canada) to determine the onset of stage II of parturition (defined as the amniotic sac visible at the vulva) and end of stage II (defined as expulsion of the calf). Two video cameras were used, one on the lateral side of the close-up pen and the other on top of the calving pens.

With regard to sample size, we estimated that 85% of the cows would calve within 12 h after the first alarm, and we proposed that the MC would have a sensitivity (**Sn**) of 90% with a 95% confidence interval with a marginal error of 10% $\left(N=1.96^{2}\times \frac{0.85\times \left(1-0.85\right)}{0.10^{2}}\right)$ (Buderer, 1996). Based on this calculation, a total of 42 cows were needed.

The data registered by the MC software were imported manually into Excel 16.0 (Microsoft, Redmond, WA) and then into Stata 17.0 (StataCorp, College Station, TX) for analysis. First, we defined 4-time intervals (2, 4, 8, and 12 h) before calving in which the first alarm was triggered. Thereafter, we proceeded to check only the first alarm received. Once we determined that the MC triggered an alarm, we looked at what the type of alarm was, 1HA or 2HA (i.e., prolonged activity in the last 2 h). The 2HA alarms occurred exactly 1 h after a 1HA alarm but not all the 1HA alarms were followed by a 2HA alarm. Also, to simplify the use of the MC, we added a third type of alarm to our analyses, a combined alarm. The latter was the first alarm after the MC fit, without considering if it was a 1HA or 2HA. Thus, we classified the first alarm events as combined, only 1HA, and only 2HA alarms.

For each of these classifications, we defined the true positive (**TP**), true negative (**TN**), false positive (**FP**), and false negative (**FN**) fractions. If a cow calved within 2, 4, 8, or 12 h after the first alarm received during the average time (66 h; Table 1) the MC was fitted, it was considered a TP, whereas if the cow did not calve within 2, 4, 8, or 12 h after that first alarm, it was considered a FP. If the MC did not generate an alarm and the cow did not calve during the average time the MC was fitted, it was considered a TN. In contrast, a FN was a cow that did not have an alarm but calved during the average time the MC was fitted (Table 1). Once the negative (TN and FN) and positive (TP and FP) fractions were defined, the Sn, specificity (**Sp**), positive predicted values (**PPV**), and negative predicted values (**NPV**) for each of the 4 time intervals were determined. Receiver operating characteristic (**ROC**) curves were generated and area under curve (**AUC**) values were calculated to measure the accuracy of MC for different time points (2, 4, 8, and 12 h) relative to calving considering only the first alarm received after the MC fit. Graphs for the Sn and Sp, and ROC-AUC curves were completed in Excel 16.0 (Microsoft, Redmond, WA).

**Table 1:** Summary of the descriptive data from the 49 animals enrolled in the Moocall (Bluebell) validation study according to the Moocall software information and the sensitivity and specificity analysis criteria

Event^1^		Value
Number of animals enrolled		49
Number of animals included in the analysis		36
Number of animals with an alarm recorded		28
Average time between Moocall fit and calving, h (mean ± SD)		66 ± 56
Average time from Moocall fit to first alarm, h (mean ± SD)		49.6 ± 53.7
Average time from Moocall fit to second alarm, h (mean ± SD)		52.7 ± 49.9
Number of alarms/animal (mean ± SD)		2.7 ± 2.3
Number of 1HA alarms		10
Number of 2HA alarms		18
Number of animals with no alarm registered^2^		8
Number of Moocall drops		29
Number of false negative and positive alarms 12 h relative to calving	Positive	5
	Negative	5
Number of false negative and positive alarms 8 h relative to calving	Positive	7
	Negative	5
Number of false negative and positive alarms 4 h relative to calving	Positive	10
	Negative	5
Number of false negative and positive alarms 2 h relative to calving	Positive	13
	Negative	5

1 1HA = high tail activity in the previous hour; 2HA = high tail activity in the previous 2 h.

2 Animals that did not have an alarm and calved.

A total of 29 MC drops occurred in 21 (42%) cows out of the 49 cows enrolled in the study. We excluded 13 animals from the analysis as they had 3 or more MC drops (n = 6), a swollen tail (n = 3), or the MC device was lost (n = 4). Therefore, data from 36 animals were analyzed (Table 1). The average (± SD) time from MC fit to the onset of stage II of calving was 66 ± 56 h. On average, 1HA alarm (n = 28 cows) was registered 49.6 ± 53.7 h after the MC was fitted, and on average, 17 h before the onset of stage 2 of calving. The 2HA alarm (n = 18 cows) was registered on average 52.7 ± 49.9 h after the MC was fitted, and on average, 13 h before the onset of stage 2 of calving. The average number of alarms (1HA and 2HA) per cow before the onset of stage II of calving was 2.7 ± 2.3. Regarding the MC activity, 8 cows did not register an alarm from MC fit to calving and, when classifying the type of first alarm, 10 cows only had 1HA alarm and 18 cows had 1HA alarm followed by a 2HA alarm; therefore, they were classified as 2HA alarm cows.

Results of the diagnostic test at each time interval before calving are presented in Table 2. The MC alarm had the highest Sn and Sp at 12 h before calving. The value of Sn, Sp, PPV, and NPV for combined alarms was 72.2%. For the 2HA alarms, the Sn was 50.0%, Sp was 93.7%, PPV was 90.1%, and NPV was 60.0%. The 1HA alarms had the poorest performance, with Sn, Sp, PPV, and NPV being 15.8%, 76.4%, 42.9%, and 44.8%, respectively. At 12 h before calving, the predicted ability was higher for the combined alarms (AUC: 0.77) and the 2HA alarms (AUC: 0.75) compared with the 1HA alarms (AUC: 0.43; Figure 1A).

**Table 2:** Summary of the predictive performance from the 36 animals that were not excluded during the Moocall (Bluebell) validation study according to the Moocall software information and the sensitivity and specificity analysis criteria

Diagnostic test^2^	Time interval before calving^1^			
	2 h	4 h	8 h	12 h
Sensitivity				
1HA	5.8	5.8	11.1	15.8
2HA	25.0	36.8	47.4	50.0
1HA + 2HA	50.0	61.5	68.8	72.2
Specificity				
1HA	68.4	68.4	73.2	76.4
2HA	65.6	76.5	88.2	93.7
1HA + 2HA	50.0	56.5	65.0	72.2
PPV^3^				
1HA	14.3	27.8	28.6	42.9
2HA	36.6	63.6	81.8	90.1
1HA + 2HA	27.8	44.4	61.1	72.2
NPV^3^				
1HA	44.8	44.8	44.8	44.8
2HA	52.0	52.0	60.0	60.0
HA + 2HA	72.2	72.2	72.2	72.2

1 Start of stage II of calving.

2 1HA + 2HA represents the combination of the 2 alarms, 1HA and 2HA. 1HA = high tail activity in the previous hour; 2HA = high tail activity in the previous 2 h.

3 PPV = positive predictive value; NPV = negative predictive value.

**Figure 1:**
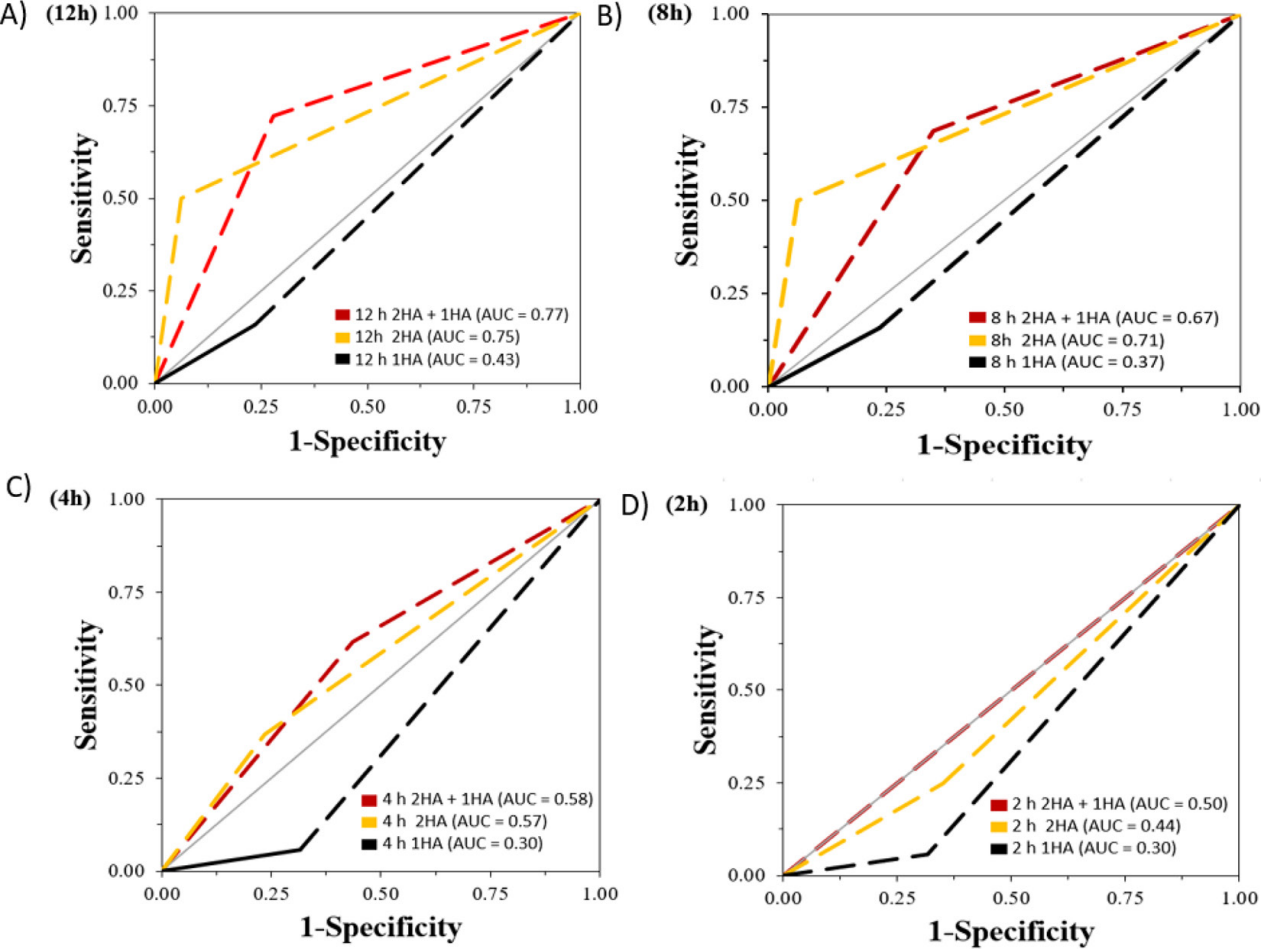
Moocall (Bluebell) accuracy to discriminate between calving and no-calving events based on receiver operator characteristic curves and area under the curve values for 36 Holstein dairy cows. (A) represents values from combined alarms (1HA + 2HA), and separately 1HA alarms and 2 HA alarms at 12 h before calving. (B) represents values from combined alarms (1HA + 2HA), and separately 1HA alarms and 2 HA alarms at 8 h before calving. (C) represents values from combined alarms (1HA + 2HA), and separately 1HA alarms and 2 HA alarms at 4 h before calving. (D) represents values from combined alarms (1HA + 2HA), and separately 1HA alarms and 2 HA alarms at 2 before calving. 1HA = high tail activity in the previous hour; 2HA = high tail activity in the previous 2 h.

At 8 h relative to calving, the Sn for the combination of 2 alarms was 68.8%, whereas the Sp, PPV, and NPV were 65.0%, 61.1%, and 72.2%, respectively. For the 2HA alarms, the Sn was 47.4%, Sp was 88.2%, PPV was 81.8%, and NPV was 60.0%. Regarding the 1HA alarms, the Sn, Sp, PPV, and NPV were 11.1%, 73.2%, 28.6%, and 44.8%, respectively. When evaluating the AUC, the predicted ability was higher for 2HA alarms (AUC = 0.71) and the combined alarms (AUC = 0.67) compared with the 1HA (AUC = 0.37; Figure 1B).

At 4 h relative to calving, the combined alarms achieved a Sn of 61.5%, whereas the Sp, PPV, and NPV for the combined alarms were 56.5%, 44.4%, and 72.2%, respectively. In contrast, for 2HA alarms the Sn was 36.8%, Sp was 76.5%, PPV was 63.6%, and NPV was 52.0%. For 1 HA alarms, the Sn, Sp, PPV, and NPV were 5.8%, 68.4%, 27.8%, and 44.8%. The AUC for 2HA alarms and the combined alarms were similar (0.57 and 0.58, respectively), whereas for 1HA alarms the AUC was 0.30 (Figure 1C).

At 2 h relative to calving, the Sn was 50.0% for combined alarms, with the Sp, PPV, and NPV being 50.0%, 27.8%, and 72.2%, respectively. For 2HA alarms, the Sn was 25.0%, Sp was 65.5%, PPV was 36.6%, and NPV was 52.0%. Regarding 1HA alarms, the Sn, Sp, PPV, and NPV were 5.8%, 68.4%, 14.3%, and 44.8%, respectively. Although the combined alarms had a higher predicted ability than those analyzed separately, none of the AUC values exceeded 0.50 (Figure 1D).

Enhanced calving monitoring and assistance are essential for identifying difficult calvings and ensuring prompt perinatal care (Mee, 2008). However, monitoring animals close to calving is a constant challenge on dairy farms (Borchers and Bewley, 2015). In this study, we evaluated the ability of an automated tail movement sensor device called Moocall to predict calving and found that information received from the device may not be sufficient to predict calving on its own.

We evaluated the performance of 3 alarms: combined, 1HA, and 2 HA. Although the highest Sn at 12 h relative to calving was obtained when considering the combined alarms, the PPV was lower than that of the 2HA alarm. This suggests that the 2HA alarm was more accurate in predicting TP individuals at 12 h relative to calving. Similarly, Voß et al. (2021) found that when alarms are combined, there is a higher Sn at 12 h relative to calving, but the PPV is low compared with 2HA alarms. Thus, it is likely that 2HA alarms are more indicative of imminent calving as they detect prolonged tail activity within a 2-h period. To further support this, Horváth et al. (2021) reported that animals having a 2HA alarm had 14.5 times higher chance of calving than animals having only 1HA alarms using an interval of 4 h from alarm to calving.

Our initial hypothesis was that 2HA alarms were more indicative of eminent calving because they indicate a persistence of tail activity compared with 1HA; however, as this was not the case in our study, we decided to combine the alarms. This may offer a more practical way to interpret the information from the MC because the user does not have to check for the type of alarm, which could be confusing. In our study, at 12 h before calving 2HA alarms had a Sp of 93.8%, whereas the combined alarms had a Sp of 72.2%, and the 1HA alarm was 76.5%. These differences are important because a high Sp indicates a low FP rate, which in practical terms will reduce unnecessary visits to the calving pen. However, at 12 h the Sn for 2HA alarms was 50.0%, indicating a high FN fraction, meaning 50.0% of the animals calving within 12 h after the first 2HA alert will not be detected by the MC. Thus, using only the 2HA alarms to predict calving within 12 h during the day could be beneficial, but relying only on this alarm during nighttime could negatively affect calving management because the device may not alert to the onset of parturition for many cows.

From 12 to 2 h before calving, the decrease in diagnostic accuracy of all the alarms was pronounced. The reduction in the predictive ability of the MC as the timeframe shortens is a common finding in other studies evaluating this device (Giaretta et al., 2021; Horváth et al., 2021; Voß et al., 2021; Górriz-Martín et al., 2022). Giaretta et al. (2021) reported a Sn of 100% and Sp of 95% for calving prediction within a 24-h window, but a Sn of 94% and Sp of 77% in a time window of 3 h (Giaretta et al., 2021). Similarly, another validation study reported Sn and Sp declining from 75% to 19% and 96% to 63%, respectively, from 12 to 2 h relative to calving (Voß et al., 2021). Contrary to our results, Voß et al. (2021) found an increase in NPV as the time relative to calving decreased from 12 to 2 h for 2HA alarms (89% vs. 97%) and 1HA alarms (86% vs. 97%). The latter is likely due to the prevalence of a calving event at each timeframe evaluated. When the prevalence of a calving event decreases, the NPV increases and vice versa for PPV (Dooho et al., 2014). For example, at 2 h relative to calving, Voß et al. (2021) reported a true prevalence (the proportion of all cows with a MC at a specific timeframe who are actually positive) of 4.0%; in contrast, we had a true prevalence of 44.0%. The latter explains why it is important to use Sn and Sp as measures of a test's performance for comparison among studies because the predictive values will vary depending on the prevalence in a population (Dooho et al., 2014).

An AUC >0.75 is considered clinically acceptable when validating the accuracy of a test or device (Fan et al., 2006). Based on this, in our study, only the combined and 2HA alarms at 12 h relative to calving were clinically acceptable to discriminate between a no-calving event and a calving event. Unfortunately, due to the limited published research on MC and the different methodologies used, we were not able to compare our AUC values with other studies. We recommend calculating test characteristics, such as AUC, in future studies using the MC to better understand its accuracy (McGrath et al., 2017).

Other wearable devices used for measuring feeding, activity, and temperature have been tested for calving. Ouellet et al. (2016) tested the predictive ability of 3 different automated monitoring devices to detect calving: an intravaginal temperature device, an ear tag for measuring rumination, and a pedometer. At 12 h before calving when considering the 3 devices, the Sn ranged from 39.0% to 69.0%, whereas the Sp ranged from 55.0% to 69.0% (Ouellet et al., 2016). In addition, all devices had an AUC <0.75 at 12 h precalving (Ouellet et al., 2016). However, unlike the MC, these devices can also be used to alert about health events and estrus, which improves their versatility and make them a more attractive for investment (Borchers and Bewley, 2015). Alternatively, combining the MC data with calving signs or with data from other automatic devices could offer valuable information to farmers in monitoring the onset of calving (Rutten et al., 2017; Giaretta et al., 2021; Górriz-Martín et al., 2022). A combined statistical model using 2HA alarms and additional traits as predictors, such as pelvic ligament relaxation, tail tip flexibility, or behavior, performed better than the MC alarms alone or statistical models without MC alerts (Górriz-Martín et al., 2022). Additionally, as Giaretta et al. (2021) suggested, using the MC in combination with rumination and activity parameters could improve calving predictability.

A limitation of this study was the number of animals excluded due to MC drops and lost devices, as well as swollen tails, which reflect an animal welfare concern. Voß et al. (2021) also reported a high level of MC drops where 557 reattachments were required among the 180 enrolled dairy cows, resulting in a total of 4 MC attachments per animal. Without investigators performing hourly observations 24 h/d, many of the MC devices in that study would likely have been lost. Furthermore, a German study reported that 58% of the animals dropped the device when it was applied 4 d before the expected due date (Górriz-Martín et al., 2022), a figure not dissimilar to that recorded here (43%).

We evaluated the applicability of an automated tail movement sensor device to predict calving time in dairy cows. Although the MC alone might not be sufficiently reliable to accurately predict calving, information derived from the MC could be combined with producer observations of onset of calving or data from other automated sensors to improve accuracy of prediction. However, the MC may benefit from exploring how to prevent device drops and damage to the tail.
